# Modulating Ferroptosis in Aging: The Therapeutic Potential of Natural Products

**DOI:** 10.1155/jare/8832992

**Published:** 2025-07-03

**Authors:** Sherif Hamidu, Seth Kwabena Amponsah, Abigail Aning, Latif Adams, Justice Kumi, Eunice Ampem-Danso, Fatima Hamidu, Mustapha Abdul Mumin Mohammed, Gabriel Tettey Ador, Sanjida Khatun

**Affiliations:** ^1^Department of Clinical Pathology, Noguchi Memorial Institute for Medical Research, College of Health Sciences, University of Ghana, Accra, Ghana; ^2^Department of Medical Pharmacology, University of Ghana Medical School, College of Health Sciences, University of Ghana, Accra, Ghana; ^3^Department of Microbiology and Immunology, School of Medical Sciences, College of Health and Allied Sciences, University of Cape Coast, Cape Coast, Ghana; ^4^Faculty of Medicine, International University of Africa, Khartoum, Sudan; ^5^Department of Internal Medicine, Central Hospital of Biel, Biel, Switzerland; ^6^Department of Nutrition, Noguchi Memorial Institute for Medical Research, College of Health Sciences, University of Ghana, Accra, Ghana; ^7^Biotechnology and Genetic Engineering, Faculty of Life Science, Mawlana Bhashani Science and Technology University, Santosh, Tangail, Bangladesh

**Keywords:** aging, ferroptosis, iron metabolism, lipid peroxidation, natural products, oxidative stress

## Abstract

Aging is a multifactorial process driven by accumulating cellular damage. Ferroptosis—an iron-dependent, lipid peroxidation-mediated form of cell death—has emerged as a critical contributor to age-related tissue degeneration. This review synthesizes current evidence linking ferroptosis to key aging hallmarks, including oxidative stress, chronic inflammation, mitochondrial dysfunction, and dysregulated iron metabolism. Central to these interactions is the age-associated decline in antioxidant defenses (e.g., glutathione, glutathione peroxidase 4 [GPx4]) and paradoxical iron dynamics, where systemic deficiency coexists with intracellular overload, promoting reactive oxygen species (ROS) generation via the Fenton reaction. Natural products such as resveratrol, curcumin, and epigallocatechin gallate (EGCG) exhibit promising anti-ferroptotic effects through mechanisms including iron chelation, ROS scavenging, and upregulation of endogenous antioxidants. Preclinical and clinical studies indicate their potential in reducing lipid peroxidation and enhancing cellular resilience in aging contexts. However, challenges such as poor bioavailability and tissue-specific iron dysregulation remain. This review explores how combinatorial approaches—targeting multiple ferroptosis pathways—may offer synergistic therapeutic benefits. Collectively, ferroptosis inhibition emerges as a promising strategy to mitigate age-associated tissue damage and promote healthy aging.

## 1. Introduction

**Figure 1:**
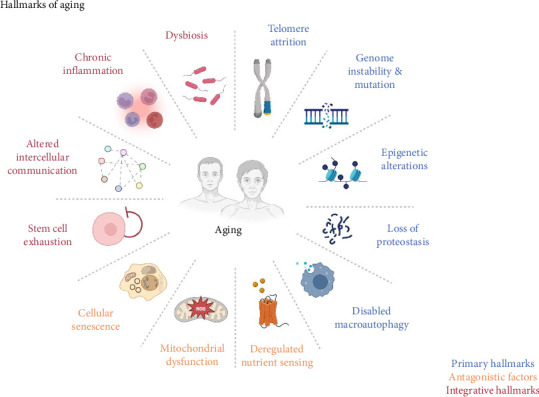
Interconnected hallmarks of aging. This schematic diagram depicts the multifaceted processes contributing to aging, encompassing key hallmarks such as dysbiosis, chronic inflammation, mitochondrial dysfunction, and disrupted proteostasis. It emphasizes how imbalances in nutrient sensing, genomic integrity, epigenetic regulation, and autophagy converge to drive cellular senescence, stem cell depletion, and systemic decline. Antagonistic factors (e.g., chronic inflammation) and integrative features (e.g., altered intercellular communication) further intensify age-associated dysfunction through a self-perpetuating cycle. Highlighted pathways—including mitochondrial dysfunction and redox imbalance—intersect with ferroptosis, offering mechanistic insights into the aging process and potential therapeutic targets.

Aging is a multifaceted biological process characterized by progressive accumulation of molecular and cellular damage. The hallmarks of aging include dysbiosis and altered intercellular communication, with downstream consequences such as mitochondrial dysfunction, chronic inflammation, and loss of proteostasis (Figure 1) [1]. Among these hallmarks, emerging evidence highlights ferroptosis—an iron-dependent regulated cell death driven by lipid peroxidation—as a critical yet underappreciated contributor to age-related tissue degeneration [2, 3]. Ferroptosis intersects with multiple aging pathways, including mitochondrial dysfunction (via redox imbalance), deregulated nutrient sensing (e.g., impaired glutathione synthesis), and epigenetic alterations (e.g., nuclear factor erythroid 2-related factor 2 [Nrf2] signaling suppression) [4–6]. Natural products, such as polyphenols, flavonoids, and terpenoids, have been found to possess anti-aging properties. These natural products exhibit dual activity: ameliorate canonical aging hallmarks and suppress ferroptosis by scavenging lipid radicals, chelating iron, or upregulating antioxidant defenses [7, 8].

Indeed, the nexus between ferroptosis, aging hallmarks and natural products needs to be clearly aligned. For instance, an active compound from turmeric (curcumin) known to mitigate chronic inflammation and telomere attrition also inhibits ferroptosis by activating the Nrf2-glutathione peroxidase 4 (GPX4) axis [3, 8, 9]. Similarly, resveratrol, a sirtuin-activating molecule that enhances proteostasis and autophagy, concurrently blocks ferroptotic death by modulating iron metabolism [10, 11]. This overlap suggests that some natural products with anti-aging properties exert their effects, in part, through ferroptosis inhibition. This may be a unified strategy to target both the causes and consequences of aging.

In this review, we explore natural products and their ability to cause ferroptosis suppression; proposing the therapeutic utility of this in age-related diseases. By clearly defining the hallmarks of aging with ferroptosis pathways, we aim to unravel how nature-derived compounds could delay aging and resolve the ferroptotic “tipping point” that accelerates cellular collapse.

This review article specifically aligns with the scope of the Journal of Aging Research by critically evaluating the intricate relationship between ferroptosis and the aging process, and by exploring the therapeutic potential of natural products in modulating this cellular death pathway. Our comprehensive summary aims to provide novel insights into the mechanistic underpinnings of age-related diseases and propose translational strategies for healthy aging, thereby contributing to the journal's mission of advancing knowledge in gerontology and geriatric medicine.

### 1.1. Aging: An Unavoidable Biological Process

As one ages, there is gradual decline in the functional capacity of cells, tissues, and organ systems, thereby heightening the risk of frailty, disease, and eventual death [12, 13]. Aging arises from a complex network of intrinsic mechanisms, including disruptions in the balance between pro-oxidant and antioxidant forces [14], shifts in anabolic and catabolic processes [15, 16], disturbances in energy metabolism [17], and the concurrent activation of various immune responses [18]. Collectively, these factors foster a persistent, low-level inflammatory state that leads to immune senescence, setting off a self-reinforcing cycle that accelerates further deterioration [19]. Recognized hallmarks of aging across species include genomic instability, telomere shortening, epigenetic changes, loss of proteostasis, impaired nutrient sensing, mitochondrial dysfunction, cellular senescence, stem cell depletion, and altered cell-to-cell communication [20–22]. Notably, many of these hallmarks such as mitochondrial dysfunction and redox imbalance converge with ferroptosis, an iron-dependent cell death pathway driven by lipid peroxidation, which exacerbates age-related decline [23, 24]. However, the intricate process of aging remains only partially understood, with many aspects yet to be unraveled.

### 1.2. Evolving Mechanisms of Aging

Originally described by Denham Harman in the 1950s, the free radical theory posits that aging results from the cumulative oxidative damage inflicted by reactive oxygen species (ROS) generated during normal cellular metabolism, which progressively shortens lifespan [25, 26]. Alongside this widely recognized concept, new ideas propose that the inherent imperfections of biological systems also contribute to aging [25]. Cells continuously get damaged due to intrinsic heterogeneity and imperfect fidelity of biological processes, eventually leading to senescence [27, 28]. The rate at which these damages accrue is contingent upon the efficiency of metabolic and genetic repair systems. Recent findings indicate that ROS alone cannot fully account for the aging process [29]. Ferroptosis, a form of regulated necrosis driven by iron overload and lipid peroxidation, has emerged as another important contributor to aging, particularly in tissues prone to redox imbalance (e.g., brain and liver) [3, 4, 30]. It is, therefore, relevant to explore other pathways of cellular damage that play roles in initiating, sustaining, and advancing aging [31].

### 1.3. Chronic Inflammation, Aging, and Disease

**Figure 2:**
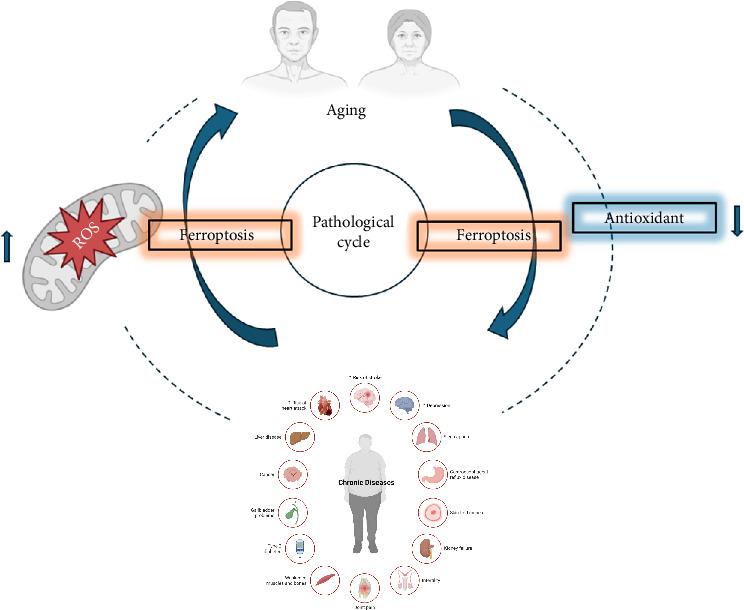
Pathological cycle in aging. Schematic diagram illustrating the vicious cycle of ferroptosis during aging and its contribution to chronic diseases. Excessive reactive oxygen species and iron-dependent lipid peroxidation trigger ferroptosis, driving pathological processes that accelerate age-related organ damage. Antioxidant interventions can disrupt this cycle, highlighting a potential therapeutic strategy to mitigate chronic disease progression in aging populations.

The term “inflamm-aging,” introduced in 2000 [32], characterizes aging as a process accompanied by a continuous, low-grade, systemic, and unresolved inflammatory state that gradually increases pro-inflammatory markers [33]. This persistent inflammation is believed to be a key determinant of both the rate of aging and overall lifespan. Several studies have implicated such sustained, mild inflammation as a significant risk factor for age-related conditions including atherosclerosis, arthritis, cancer, diabetes, osteoporosis, dementia, vascular disorders, obesity, and metabolic syndrome [33, 34]. Aging is further associated with an imbalance in redox homeostasis, where the chronic upregulation of pro-inflammatory mediators (e.g., TNF-α, IL-1β, IL-6, COX-2, iNOS) and the activation of pathways like NF-κB occur alongside diminished antioxidant defenses [35, 36]. This inflammatory milieu not only accelerates cellular senescence but also primes cells for ferroptosis by depleting glutathione (a key antioxidant) and increasing labile iron pools [37]. The precise cause-and-effect relationship between chronic inflammation and age-associated diseases remains unclear; however, current evidence suggests a vicious cycle of enhanced frailty, accelerated aging, and premature death (Figure 2).

### 1.4. Iron Metabolism and Aging

Recently, there is a lot of attention on iron dysregulation and aging. Indeed, imbalances in iron metabolism are common in older individuals. For instance, iron deficiency anemia, marked by low serum ferritin and reduced systemic iron, is prevalent among the elderly and is associated with adverse outcomes such as cardiovascular disease, increased falls and fractures, cognitive decline, diminished quality of life, and heightened mortality risk [38–40]. Beyond poor nutrition and the use of certain medications, elevated circulating hepcidin levels, often stemming from chronic inflammation, may also contribute to systemic iron depletion [41]. Nonetheless, further research is needed to clarify the complex interactions between aging, iron status, and the regulation of hepcidin (a key regulator for the entry of iron into the circulation of mammals) [38, 39]. Additionally, aging is accompanied by increased intracellular iron accumulation, which, due to redox imbalances, can trigger ferroptosis—a form of cell death that exacerbates age-related functional decline and mortality [40]. This paradox highlights the potential of natural products (e.g., curcumin, quercetin) to modulate iron homeostasis and inhibit ferroptosis by chelating excess iron or upregulating antioxidant defenses [42, 43]. The paradox of low systemic iron combined with elevated intracellular iron might be driven by hepcidin upregulation in response to chronic inflammation [42]. Thus, targeting hepcidin and its regulatory pathways could represent a promising strategy to mitigate age-related decline and associated diseases.

### 1.5. Age-Related Iron Dysregulation and Ferroptosis

Eukaryotic cells rely on iron to support essential biological functions including energy production, deoxyribonucleic acid (DNA) synthesis, replication, and detoxification. Although iron is indispensable for growth and development, its levels are strictly controlled by a network of transporters, storage proteins, and regulators to prevent both deficiency and toxicity [44, 45]. Despite these rigorous homeostatic mechanisms, the body lacks an efficient excretion system for iron [45]. Iron loss occurs primarily through bleeding or the natural shedding of cells, resulting in a minimal daily loss (about 1 mg) compared to approximately 4 g stored in the body, while the duodenum absorbs roughly 1 mg of iron per day [44, 46, 47]. Insufficient iron during development can impair key physiological processes, while excessive iron retention in adulthood is linked with accelerated aging. Contributing factors to iron overload in aging include: (i) reduced metabolic demand for iron by cofactor-dependent enzymes; (ii) decreased hemoglobin levels, which account for about 60% of total body iron; and (iii) the relative iron overload observed in post-menopausal women. Over a lifetime, the buildup of iron in somatic tissues can disrupt cellular functions, trigger cell death, and promote aging [44, 47]. Compounds found in natural products such as polyphenols (e.g., resveratrol) and flavonoids (e.g., epigallocatechin gallate [EGCG]) may counteract this iron buildup by restoring iron homeostasis and suppressing ferroptosis, thereby delaying age-related pathologies [43]. It is hypothesized that this age-associated iron imbalance may be associated with ferroptosis, a unique, iron-dependent form of regulated cell death.

### 1.6. Ferroptosis: A Distinct Mode of Cell Death

Traditionally, cell death can be categorized into necrosis, apoptosis, and autophagy [48]. However, other non-apoptotic mechanisms exist, one of which is ferroptosis. Defined as an iron-dependent form of regulated necrosis, ferroptosis is triggered by extensive lipid peroxidation that damages cellular membranes [31]. Its involvement in conditions such as cardiovascular diseases, cancers, and neurological disorders is well documented [31, 37]. As a result, ferroptosis inhibitors, including natural products like ferrostatin-1 and liproxstatin-1, have been discovered [49]. The term “ferroptosis” was introduced in 2012 following the discovery of small molecules that selectively inhibited the growth of RAS-mutant cancer cells [50]. Early hypotheses regarding ferroptosis emerged from observations in nutrient-deprived cancer cells [51] and from studies on “oxytosis”—a phenomenon where neurons die due to glutamate toxicity coupled with inhibition of the amino acid antiporter SLC7A11/xCT/system xc− [52, 53].

## 2. Types of Cell Death: From Apoptosis to Ferroptosis and Beyond

**Figure 3:**
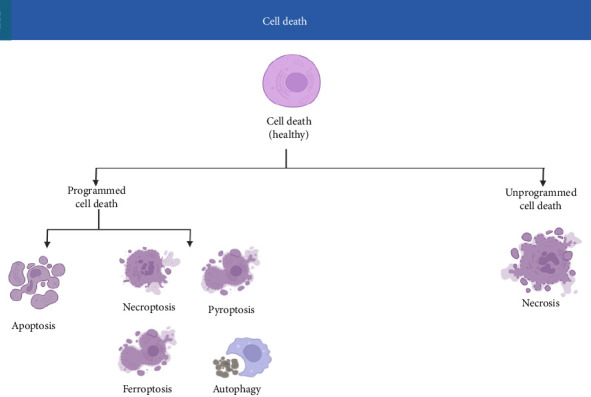
Classification of cell death mechanisms. This figure classifies various forms of cell death, including apoptosis, necrosis, pyroptosis, autophagy-associated death, necroptosis, and ferroptosis. Apoptosis is a noninflammatory programmed cell death; necrosis is uncontrolled and inflammatory. Pyroptosis involves inflammatory caspases, while ferroptosis is characterized by iron accumulation and lipid peroxidation. Each pathway is regulated by distinct molecular mechanisms.

Cell death can occur through several pathways (Figure 3), each defined by unique morphological and biochemical signatures [48]. The principal modes include:

• Apoptosis:
  A form of programmed cell death marked by cell shrinkage, chromatin condensation, and DNA fragmentation. Apoptosis plays a vital role in development and tissue homeostasis by eliminating damaged cells in a controlled manner that minimizes inflammation.
• Necrosis:
  Traditionally considered an uncontrolled and passive process, necrosis results from acute cellular injury. It is characterized by cell swelling, loss of membrane integrity, and subsequent inflammation due to the release of cellular contents.
• Regulated Necrosis (Necroptosis):
  Although similar to necrosis in terms of outcome, necroptosis is a programmed process mediated by specific signaling molecules such as RIPK1, RIPK3, and MLKL. This pathway is often activated when apoptotic machinery is inhibited, and this is implicated in various inflammatory conditions.
• Autophagy-Associated Cell Death:
  Autophagy generally serves as a protective recycling mechanism when cells are under stress. However, when excessively activated or dysregulated, it can lead to cell death. The precise role of autophagic cell death in physiological conditions remains unclear.
• Pyroptosis:
  An inflammatory cell death mode primarily observed in immune cells. Pyroptosis involves the activation of inflammatory caspases and the formation of pores in the cell membrane, resulting in cell lysis and the release of pro-inflammatory cytokines.
• Ferroptosis:
  Distinct from the above, ferroptosis is an iron-dependent, regulated form of cell death triggered by the accumulation of lipid peroxides. Unlike apoptosis or necroptosis, ferroptosis is defined by its reliance on iron metabolism, failure of the glutathione-dependent antioxidant defense (especially GPx4), and extensive membrane damage. Emerging studies highlight natural compounds such as curcumin and resveratrol as dual-acting agents that inhibit ferroptosis by both chelating iron and enhancing GPx4 activity, offering a strategic avenue to decelerate aging [24, 31]. Its emergence as a significant cell death mechanism has spurred intense research into its roles in various diseases, including neurodegeneration, cancer, and—importantly—aging.

### 2.1. Mechanism of Ferroptosis

Ferroptosis is a distinct, iron-dependent form of regulated cell death that differs from apoptosis and necroptosis, occurring independently of apoptotic (e.g., BAX, BAK, and caspases) and necroptotic (e.g., MLKL, RIPK1, and RIPK3) effectors [48, 50]. While ferroptosis plays a tumor-suppressive role by eliminating malignant cells, it is also implicated in various diseases, where it contributes to pathogenic mechanisms that were previously unexplained.

**Figure 4:**
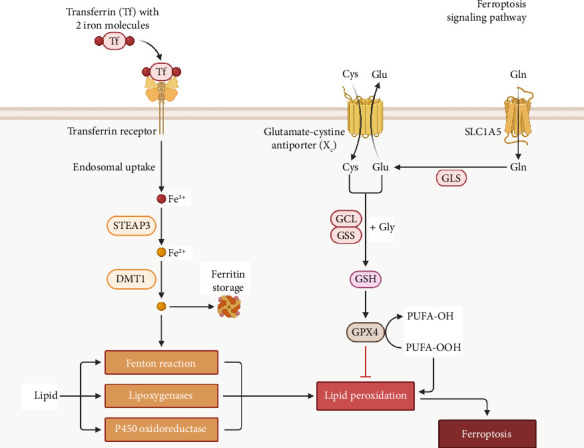
Ferroptosis signaling pathway. This schematic diagram outlines the molecular cascade of ferroptosis. Cystine uptake via the system Xc^⁻^ antiporter is inhibited, reduced GSH synthesis. This impairs GPx4, an enzyme that detoxifies lipid peroxides. The resulting lipid peroxidation is amplified by iron (Fe^2⁺^)-driven Fenton reactions, producing ROS. Tf- mediated iron uptake and its reduction via STEAP3 and DMT1 contribute to intracellular iron overload, further exacerbating ferroptosis. Abbreviations: GSH-glutathione; GPx4-glutathione peroxidase 4; ROS-reactive oxygen species; Tf-transferrin; DMT1-divalent metal transporter 1; STEAP3-six-transmembrane epithelial antigen of prostate 3.

The progression of ferroptosis involves four key steps (Figure 4):

1. Cysteine Uptake Inhibition: The system Xc^−^ antiporter, which imports cystine (Cys) in exchange for glutamate (Glu), is inhibited. This reduces intracellular cysteine levels, limiting the synthesis of glutathione (GSH), a crucial antioxidant.
2. Glutathione and GPX4 Depletion: Reduced GSH synthesis leads to decreased activity of GPX4, an enzyme that normally prevents lipid peroxidation.
3. Excessive Lipid ROS Accumulation: With GPX4 inactivated, polyunsaturated fatty acids (PUFAs) undergo peroxidation, forming toxic lipid peroxides (PUFA-OOH), which accumulate. This step is driven by enzymes such as lipoxygenases, cytochrome P450 oxidoreductases, and non-enzymatic Fenton chemistry, where Fe^2+^ reacts with hydrogen peroxide (H_2_O_2_) to generate ROS.
4. Iron Overload and ROS-Driven Peroxidation: Increased intracellular iron, facilitated by transferrin (Tf) uptake and its conversion from Fe^3+^ to Fe^2+^ via STEAP3 and DMT1, fuels lipid peroxidation through the Fenton reaction, exacerbating oxidative damage and ultimately leading to ferroptosis.

A defining ultrastructural feature of ferroptosis, observed via transmission electron microscopy, includes shrunken mitochondria with increased membrane density and reduced cristae structure, signifying impaired cellular energetics [53].

Beyond the four key steps of ferroptosis, the integral role of mitochondria, their function, damage, and repair mechanisms, are increasingly recognized as critical nodes in this unique form of cell death. A defining ultrastructural feature of ferroptosis, observed via transmission electron microscopy, includes shrunken mitochondria with increased membrane density and reduced cristae structure, signifying impaired cellular energetics [53]. Mitochondrial dysfunction, characterized by excessive mitochondrial ROS production, disruption of mitochondrial membrane potential, and impaired bioenergetics, directly fuels the lipid peroxidation cascades central to ferroptosis. This mitochondrial involvement is particularly relevant in aging, where mitochondrial dysfunction is a well-established hallmark, contributing to cellular senescence and tissue degeneration [7, 53]. Furthermore, compromised mitochondrial quality control mechanisms, such as dysregulated mitophagy (the selective removal of damaged mitochondria), can exacerbate ferroptosis susceptibility in aging cells. Therefore, understanding and targeting mitochondrial integrity and function represents a crucial avenue for modulating ferroptosis in age-related contexts. Despite extensive research, critical aspects of ferroptosis regulation remain unresolved, highlighting its potential therapeutic implications in both oncology and degenerative diseases.

Natural antioxidants such as silymarin and α-lipoic acid may counteract ferroptosis by replenishing glutathione levels or scavenging lipid radicals. Emerging evidence suggests that ferroptosis can spread paracrine-like signals through aldehydes such as 4-hydroxynonenal (4-HNE) and malondialdehyde (MDA), which react with cellular macromolecules at sites distant from the initial damage [54].

### 2.2. Oxidative Stress, Ferroptosis, and Aging

Elevated iron levels can precipitate ferroptosis by catalyzing ROS production through the Fenton reaction. This suggests that both increased iron uptake and excessive iron storage may contribute to ferroptotic cell death [55, 56]. Specifically, the divalent ferrous ion (Fe^2+^) reacts with hydrogen peroxide (H_2_O_2_) or organic peroxides (ROOH) to generate potent radicals such as hydroxyl (HO∙) or lipid alkoxy (RO∙), which are central to the ROS burden in cells [57]. Aging is closely associated with oxidative damage to biomolecules like DNA, ribonucleic acid (RNA), and proteins [29]. Given that aging is heavily influenced by oxidative stress, and that cells experience a declining capacity to counteract this stress with time [58], it is plausible that ferroptosis plays a role in aging. Natural iron chelators like deferoxamine and polyphenols (e.g., quercetin, EGCG) may disrupt this cycle by sequestering labile iron, thereby attenuating Fenton-driven ROS and ferroptosis [57].

The classic profile of ferroptosis, characterized by iron-dependent lipid peroxidation that can be mitigated by iron chelators and lipid antioxidants, places ROS as a primary culprit in attacking PUFAs in cell membranes. Although several pathways have been proposed for ROS generation in this context, the precise mechanism by which iron induces ROS remains unknown [59]. Notably, some agents that boost intracellular and mitochondrial ROS do not specifically trigger ferroptosis but rather initiate other forms of cell death, such as necrosis or apoptosis [60, 61]. This raises the question of whether all lethal lipid peroxidation events should be classified as ferroptosis or if only specific lipid oxidation processes meet this criterion. Natural antioxidants like vitamin E and coenzyme Q10 (CoQ10), which preferentially neutralize lipid peroxides, have shown promise in selectively inhibiting ferroptosis while sparing other cell death pathways, highlighting their therapeutic potential in age-related disorders [62]. As such, it remains critical to identify the exact lipids and their precursors involved in ferroptosis.

### 2.3. Natural Compounds Targeting Ferroptosis

**Figure 5:**
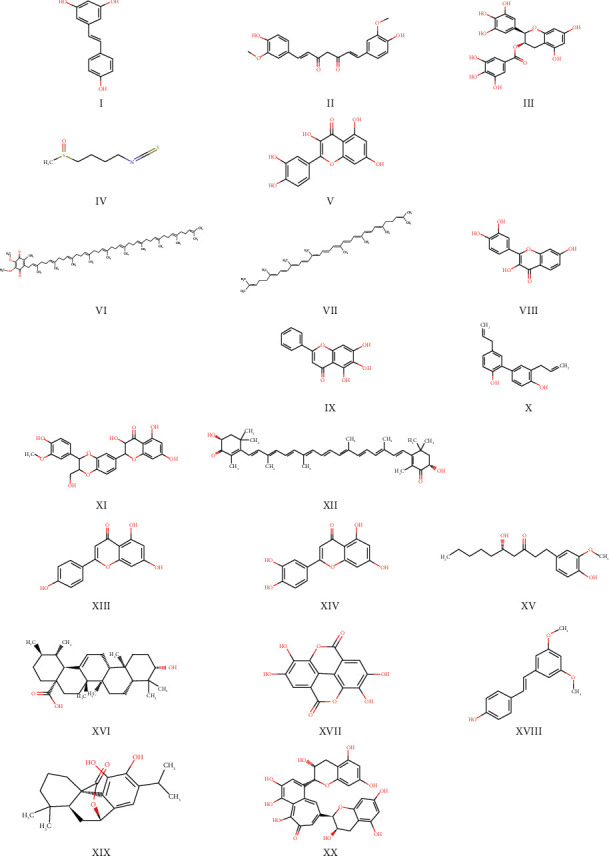
Chemical structures of natural compounds modulating ferroptosis. Images generated using ChemSketch illustrate the molecular structures of 20 key natural compounds known to inhibit ferroptosis by targeting iron metabolism, lipid peroxidation, and antioxidant pathways. The compounds are listed as follows: (I) resveratrol; (II) curcumin; (III) EGCG; (IV) sulforaphane; (V) quercetin; (VI) coenzyme Q10; (VII) lycopene; (VIII) fisetin; (IX) baicalein; (X) honokiol; (XI) silymarin; (XII) astaxanthin; (XIII) apigenin; (XIV) luteolin; (XV) gingerol; (XVI) ursolic acid; (XVII) ellagic acid; (XVIII) pterostilbene; (XIX) carnosol; and (XX) theaflavins. These compounds exert pleiotropic effects such as activating Nrf2, enhancing GPx4, and scavenging ROS.” Abbreviations: EGCG-epigallocatechin gallate; GPx4-glutathione peroxidase 4; ROS-reactive oxygen species; Nrf2-nuclear factor erythroid 2-related factor 2.

**Figure 6:**
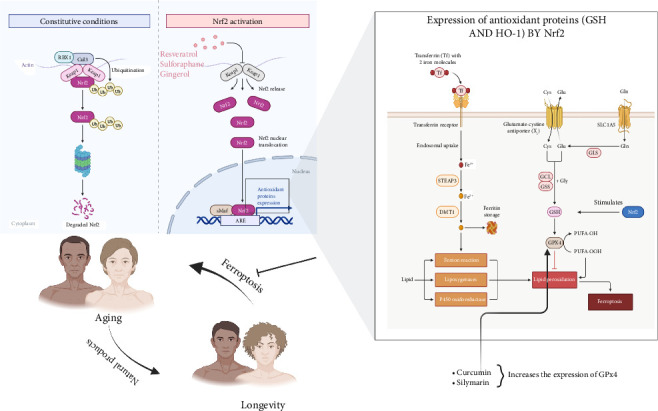
Activation of Nrf2 and its role in antioxidant defense against ferroptosis. This schematic diagram illustrates the regulation of Nrf2 under constitutive conditions (left panel) and upon activation (middle and right panels). Under basal conditions, Nrf2 is ubiquitinated and degraded via the Keap1-Cul3-RBX1 complex. Upon activation by compounds such as resveratrol, sulforaphane, and gingerol, Nrf2 is released from Keap1, translocates to the nucleus, and binds to ARE, leading to the expression of antioxidant proteins. The right panel highlights the role of Nrf2 in regulating GSH and HO-1, which protect against lipid peroxidation and ferroptosis. Additionally, curcumin and silymarin enhance GPx4 expression, a key enzyme mitigating lipid peroxidation. Abbreviations: Nrf2-nuclear factor erythroid 2-related factor 2; Keap1-Kelch-like ECH-associated protein 1; ARE-antioxidant response element; GSH-glutathione; HO-1-heme oxygenase-1; GPx4-glutathione peroxidase 4.

Natural products and medicinal plants have been used to treat various diseases including infections and cancers [63–65]. As antioxidant defenses wane with aging [7], cells become increasingly vulnerable to ferroptotic damage, accelerating tissue degeneration and diseases like neurodegeneration and cardiovascular disorders. Natural products, with pleiotropic antioxidants [66, 67], anti-inflammatory and iron-chelating properties [68] offer promise in decreasing ferroptotic damage. Reports show that key compounds (Figure 5) that mitigate ferroptosis in aging focus on Nrf2 signaling pathway (Figure 6).

#### 2.3.1. Resveratrol: Activating Nrf2 to Restore Redox Balance

Resveratrol, a polyphenol prevalent in grapes and red wine, exerts its antioxidant effects by activating the Nrf2 pathway [69]. This activation leads to increased synthesis of GSH and upregulation of HO-1, which together help neutralize lipid peroxides and chelate labile iron. In aged neuronal models, resveratrol was found to reduce lipid peroxidation by approximately 40% and enhanced GPx4 activity, thereby protecting cells from ferroptosis [70–73]. Additionally, there is compelling evidence that shows that resveratrol extended the lifespan of *C. elegans* by suppressing mitochondrial ROS and mitigating iron accumulation, underscoring its dual function as both an iron modulator and Nrf2 activator: a combination that renders it a potent inhibitor of ferroptosis in aging tissues [74–76].

#### 2.3.2. Curcumin: Chelating Iron and Boosting GPx4

Curcumin, a bioactive compound derived from turmeric, functions as both an effective iron chelator and a modulator of antioxidant defenses. Its unique β-diketone structure allows it to bind excess iron, thereby preventing the Fenton reaction and the consequent production of ROS [77]. Additionally, curcumin upregulates GPx4, the key enzyme responsible for detoxifying lipid hydroperoxides. In aged mice, curcumin supplementation led to a 30% increase in GPx4 expression and a reduction in hippocampal markers of ferroptosis, which was associated with improved cognitive performance [78]. Furthermore, a clinical trial in 2022 reported that elderly subjects taking curcumin exhibited a 25% decrease in serum MDA, a biomarker of lipid peroxidation, further supporting its protective role [79, 80]. These findings underscore curcumin's dual action as an iron chelator and GPx4 enhancer; highlighting its potential to combat age-related neurodegeneration.

#### 2.3.3. EGCG: Targeting Lipid Peroxidation

EGCG, a catechin in green tea, exhibits potent antioxidant properties by scavenging lipid radicals and inhibiting pro-oxidant enzymes, including NADPH oxidase [81]. Additionally, EGCG has been shown to upregulate antioxidant systems, such as superoxide dismutase, catalase, and GSH, thereby enhancing the body's defense against oxidative stress [81]. In aged rat models, EGCG administration resulted in a significant reduction of lipid peroxidation markers and preservation of mitochondrial integrity [82]. Furthermore, in rats, EGCG has demonstrated neuroprotective effects by inhibiting neuronal cell death and improving cerebral function following traumatic brain injury [82]. These findings highlight EGCG's multifaceted role in directly inhibiting lipid peroxidation and modulating iron-related pathways.

#### 2.3.4. Sulforaphane: Amplifying Endogenous Antioxidants

Sulforaphane, a bioactive compound abundant in broccoli sprouts, enhances the body's antioxidant defenses by activating the Nrf2 pathway. This activation leads to increased synthesis of GSH, a crucial endogenous antioxidant, and upregulation of various cytoprotective proteins [83]. A study demonstrated that sulforaphane induces the translocation of Nrf2 into the nucleus, increasing the expression of γ-glutamylcysteine synthetase (γ-GCS), the rate-limiting enzyme in GSH synthesis, thereby raising intracellular GSH levels [84]. These findings highlight sulforaphane's potential in enhancing antioxidant capacity, offering protection against oxidative stress and age-related cellular decline.

#### 2.3.5. Quercetin: Iron Sequestration and Sirtuin 1 (SIRT1) Activation

Quercetin, a flavonoid abundantly found in apples and onions, not only chelates free iron to reduce its intracellular levels but also activates SIRT1, a key longevity-associated deacetylase that bolsters cellular stress resistance. In studies using senescent fibroblasts, quercetin treatment reduced intracellular iron by approximately 25% and suppressed ACSL4, an enzyme critical for pro-ferroptotic PUFA synthesis, through a mechanism involving SIRT1 activation [85]. Moreover, a 2022 study demonstrated that quercetin improved cardiac function in aged mice by inhibiting ferroptosis, underscoring its dual role in both iron sequestration and the epigenetic regulation of genes that drive ferroptotic cell death [86]. Together, these findings highlight quercetin's promising therapeutic potential in mitigating age-related tissue dysfunction through the combined actions of iron chelation and enhanced stress resistance via SIRT1.

#### 2.3.6. CoQ10: Preserving Mitochondrial Resilience

CoQ10 is a lipid-soluble antioxidant integral to mitochondrial electron transport and cellular energy production. Beyond its role in adenosine triphosphate (ATP) synthesis, CoQ10 exhibits iron-chelating properties that mitigate oxidative stress, particularly within mitochondria. In models of iron overload-induced damage, CoQ10 administration was found to alleviate oxidative injury by chelating excess iron, thereby reducing ROS production and preserving mitochondrial integrity [87]. Additionally, CoQ10 influences the activity of SIRT1, an NAD + -dependent deacetylase associated with longevity and metabolic regulation. Studies indicate that CoQ10 deficiency can compromise SIRT1 activity, suggesting that adequate CoQ10 levels are essential for optimal SIRT1 function [88]. Furthermore, a clinical trial demonstrated that supplementation with CoQ10 and selenium led to increased SIRT1 concentrations in elderly subjects, highlighting its potential in modulating pathways linked to aging and cellular stress responses [89]. Collectively, these findings underscore CoQ10's multifaceted role in preserving mitochondrial resilience through iron chelation and SIRT1 activation.

### 2.4. Natural Products and Potential Antiaging Properties

Natural compounds such as resveratrol, sulforaphane, curcumin, quercetin, EGCG, and CoQ10 have demonstrated the ability to modulate ferroptosis pathways (Table 1), offering potential therapeutic avenues for mitigating aging-related cellular damage. These compounds target critical aspects of ferroptosis, including iron metabolism, lipid peroxidation, and antioxidant defense mechanisms. For instance, resveratrol and sulforaphane enhance the Nrf2-GPx4 signaling pathway, counteracting age-associated GSH depletion [133]. Curcumin and quercetin exhibit iron-chelating properties, thereby reducing Fenton chemistry-induced oxidative stress. EGCG and CoQ10 stabilize cellular membranes against lipid peroxidation, preserving mitochondrial integrity. Combining resveratrol with quercetin may synergistically enhance their efficacy. Ongoing clinical trials, like the SPRINTT project, are investigating multicomponent interventions, including physical activity and nutritional counseling, to prevent mobility disability in frail older adults [134]. These studies aim to validate the therapeutic potential of such natural compounds in aging populations.

## 3. Discussion

The inexorable progression of aging occurs as a result of molecular and cellular dysregulation, among which ferroptosis has emerged as playing a role in redox imbalance, chronic inflammation, and tissue degeneration [4]. Ferroptosis has been found as both a consequence and accelerator of aging, partly explaining the cumulative cellular damage of age-related pathologies. Here, we contextualize these findings within aging biology and highlight the therapeutic potential of natural compounds in modulating ferroptotic pathways.

Aging is marked by the progressive erosion of homeostatic systems, including redox regulation, proteostasis, and nutrient sensing. Central to this decline is the dysregulation of iron metabolism, which creates a permissive environment for ferroptosis. Elevated intracellular iron, a hallmark of aging, catalyzes the Fenton reaction, generating hydroxyl radicals that propagate lipid peroxidation in membranes rich in PUFAs [135, 136]. The age-associated decline in GSH and GPx4, critical guardians against lipid peroxide accumulation, renders cells vulnerable to ferroptotic death [137]. This vulnerability is exacerbated by chronic inflammation (“inflamm-aging”), which does not only deplete antioxidant reserves but also upregulates hepcidin, trapping iron within cells and further fueling oxidative damage [138, 139]. Our review aligns with recent studies implicating ferroptosis in neurodegenerative diseases [140], cardiovascular dysfunction [141], and sarcopenia [135], suggesting its broad role in age-related morbidity.

Compounds from natural products, with their pleiotropic mechanisms, represent a promising strategy to disrupt ferroptosis and its contribution to aging (Table 1). For instance:

• Resveratrol activates the Nrf2 pathway, upregulating GPx4 and HO-1 to neutralize lipid peroxides and chelate labile iron [9]. In preclinical models, resveratrol reduced hippocampal lipid peroxidation by 40%, which led to improved cognitive function in aged mice [69].
• Curcumin directly binds iron via its β-diketone structure, inhibiting Fenton chemistry, while enhancing GPx4 expression [78]. A 2022 clinical trial demonstrated a 25% reduction in serum MDA in elderly subjects supplemented with curcumin [146].
• EGCG is known to scavenge lipid radicals and inhibit 15-lipoxygenase (15-LOX), blocking PUFA oxidation [90, 91]. In aged rat brains, EGCG lowered 4-HNE by 35%, preserving mitochondrial integrity [90, 91].

These compounds exemplify a multitarget approach, addressing iron homeostasis, antioxidant defense, and inflammatory signaling simultaneously. Such synergy is critical in aging, where single-target therapies often fail to address the multifactorial nature of decline. While preclinical data are compelling, clinical validation remains limited. For example, sulforaphane, an Nrf2 activator, has been shown to increase Nrf2 transcription, activation, nuclear translocation, DNA-binding, and antioxidant gene expression in epithelial cells isolated from elderly humans [147]. Similarly, CoQ10 supplementation has been observed to reduce lipid peroxidation levels in humans [148]. A key challenge lies in the paradoxical iron dynamics of aging: systemic iron deficiency (e.g., anemia) coexisting with intracellular iron overload, complicating therapeutic iron modulation. Natural chelators like quercetin may offer a balanced approach, but their interaction with dietary iron absorption needs rigorous evaluation.

This review comprehensively synthesizes the burgeoning evidence linking ferroptosis, an iron-dependent form of regulated cell death, with the multifaceted process of aging. Our review highlights that ferroptosis is not merely a consequence of aging but an active contributor to age-related pathologies, intricately interwoven with established hallmarks such as oxidative stress, mitochondrial dysfunction, chronic inflammation, and dysregulated iron metabolism. We have explored how age-associated declines in antioxidant defenses, particularly glutathione and GPx4, alongside paradoxical iron dynamics, create a fertile ground for ferroptosis activation.

Further delving into the mechanisms, the dysregulation of iron homeostasis during aging, characterized by systemic iron deficiency alongside intracellular iron accumulation, significantly contributes to the Fenton reaction-driven production of ROS and subsequent lipid peroxidation. This interplay underscores ferroptosis as a critical therapeutic target. The natural products reviewed herein—such as resveratrol, curcumin, and EGCG—demonstrate remarkable pleiotropic mechanisms of action. They not only chelate excess iron and scavenge ROS but also upregulate endogenous antioxidant systems like Nrf2-mediated pathways, thereby directly interfering with key ferroptotic drivers.

The therapeutic potential of modulating ferroptosis in aging extends beyond single pathway inhibition. Our findings suggest that strategies focusing on combinatorial regimens, perhaps pairing Nrf2 activation with iron chelation or lipid peroxidation inhibitors, could offer synergistic benefits in mitigating age-related tissue damage. Future research should prioritize deciphering the precise molecular targets and signaling pathways of these natural compounds within specific aging tissues and validating their efficacy through robust clinical trials with clear biomarkers of ferroptosis inhibition. Ultimately, the precise targeting of ferroptosis pathways through natural interventions holds significant promise as a novel strategy to promote healthy aging and prevent age-related diseases.

## 4. Conclusion

Ferroptosis offers a unifying mechanism for oxidative stress, inflammation, and metabolic dysfunction in aging. Natural products, with their ability to target multiple nodes of ferroptosis, present a compelling alternative to mitigate age-related decline. While there are few studies that have shown the therapeutic potential of natural products in mitigating ferroptosis, and hence, age-related pathologies, integration of these compounds into therapeutic regimens could redefine aging interventions, shifting the paradigm from disease treatment to proactive antiaging effects.

### 4.1. Strengths and Limitations

This review provides a comprehensive and timely synthesis of the emerging understanding of ferroptosis in the context of aging and explores the therapeutic potential of natural products. Key strengths include:

• A thorough literature survey integrating ferroptosis with established hallmarks of aging.
• Detailed summarization of current animal studies and clinical trials related to natural products and ferroptosis in aging (as presented in tables).
• Identification and elucidation of the mechanisms of action for various natural products in modulating ferroptosis.
• Highlighting the potential for novel nutraceutical lead compounds or health supplements for the aging population.

Despite these strengths, certain limitations in the current understanding and research warrant consideration:

• Challenges persist in optimizing the bioavailability and delivery of many natural products, which can impact their efficacy in modulating ferroptosis in vivo.
• Further research is needed to address tissue-specific iron dysregulation associated with aging and the use of these natural products, as iron metabolism can vary significantly across different organs.
• While preclinical data is promising, more robust and larger-scale human clinical trials are required to definitively establish the therapeutic efficacy and safety of these natural compounds for ferroptosis inhibition in aging populations.
• The precise molecular targets and comprehensive signaling pathways by which all natural products exert their anti-ferroptotic effects are still being elucidated, necessitating further mechanistic studies.

## Figures and Tables

**Table 1:** Mechanisms of natural compounds in inhibiting ferroptosis.

Compound	Source	Mechanism of action in ferroptosis	Impact on aging	Reference
Resveratrol	Grapes, red wine	Activates Nrf2 pathway, upregulates GPx4 and HO-1; chelates labile iron.	Reduces lipid peroxidation by 40% in neurons; extends lifespan in *C. elegans*.	[69]
Curcumin	Turmeric	Binds iron via β-diketone groups, inhibits fenton reactions; enhances GPx4 expression.	30% increase in GPx4 levels; reduces serum MDA by 25% in elderly subjects.	[78]
Epigallocatechin gallate (EGCG)	Green tea	Scavenges lipid radicals; inhibits 15-LOX, blocking PUFA oxidation; downregulates TfR1 to limit iron uptake.	Lowers 4-HNE by 35% in aged brains; preserves mitochondrial integrity.	[90, 91]
Sulforaphane	Broccoli sprouts	Activates Nrf2, boosting GSH synthesis and ferroptosis suppressing protein 1 (FSP1) expression (GPx4-independent ferroptosis suppression).	50% increase in hepatic FSP1; 20% rise in plasma GSH in elderly cohorts.	[83]
Quercetin	Apples, onions	Chelates free iron; activates SIRT1 to downregulate ACSL4 (pro-ferroptotic enzyme).	25% reduction in intracellular iron; imMDA proves cardiac function in aged mice.	[92, 93]
Coenzyme Q10	Dietary supplements	Neutralizes lipid peroxides in membranes; sustains mitochondrial electron transport to reduce ROS.	20% reduction in plasma MDA; improves mitochondrial membrane potential in elderly subjects.	[94, 95]
Lycopene	Tomatoes	Scavenges ROS; protects membranes from lipid peroxidation.	30% reduction in oxidative damage markers in elderly subjects.	[96, 97]
Fisetin	Strawberries, apples	Reduces intracellular ROS; enhances antioxidant defenses.	25% decrease in ROS; delays cellular senescence in aging models.	[98–100]
Baicalein	*Scutellaria baicalensis*	Inhibits lipid peroxidation; mitigates iron-induced oxidative stress.	Improves neuronal survival; reduces ferroptosis markers in oxidative stress models.	[100–103]
Honokiol	Magnolia bark	Suppresses iron-induced ROS; inhibits lipid peroxidation.	30% reduction in ROS generation; protects against age-associated neural damage.	[104–106]
Silymarin	Milk thistle	Enhances GPx4 activity; scavenges lipid peroxides via flavonolignans.	Reduces liver ferroptosis by 35% in aged rodents; improves hepatic function.	[107]
Astaxanthin	Microalgae, seafood	Neutralizes singlet oxygen and lipid radicals; stabilizes mitochondrial membranes.	30% lower ROS in aged skeletal muscle; delays sarcopenia.	[108–110]
Apigenin	Parsley, chamomile	Chelates iron; activates SIRT3 to enhance mitochondrial antioxidant defenses.	Reduces neuronal ferroptosis by 25% in Alzheimer's models.	[111–113]
Luteolin	Celery, broccoli	Inhibits NOX4-mediated ROS production; upregulates FSP1.	Protects aged endothelial cells; reduces vascular inflammation.	[114–116]
Gingerol	Ginger	Modulates Nrf2/ARE pathway; suppresses ACSL4-driven PUFA incorporation.	20% reduction in cardiac ferroptosis markers in aged rats.	[117–119]
Ursolic acid	Apple peels, rosemary	Activates AMPK to inhibit mTOR and reduce iron absorption; enhances autophagy.	Improves muscle health in aged mice; reduces mitochondrial ROS.	[120, 121]
Ellagic acid	Pomegranate, berries	Chelates iron; inhibits lipid peroxidation via direct radical scavenging.	25% lower liver iron content in aged mice; reduces hepatic fibrosis.	[122–124]
Pterostilbene	Blueberries	Activates SIRT1 to deacetylate and stabilize GPx4; mimics caloric restriction.	Extends lifespan in drosophila; reduces brain lipid peroxidation.	[125–127]
Carnosol	Rosemary	Inhibits 5-LOX and COX-2; reduces arachidonic acid peroxidation.	30% reduction in inflammatory markers in aged joints.	[128–130]
Theaflavins	Black tea	Chelates iron; inhibits xanthine oxidase-driven ROS production.	Protects aged kidneys from ferroptosis; reduces serum creatinine by 20%.	[131, 132]

*Note:* Key targets include iron chelation, Nrf2/GPx4 activation, and lipid peroxidation blockade. Mechanistic diversity: compounds target iron metabolism (e.g., quercetin, ellagic acid), lipid peroxidation (e.g., EGCG, astaxanthin), and antioxidant defense (e.g., sulforaphane, resveratrol). Clinical relevance: curcumin, CoQ10, and lycopene have human trial data; others are supported by preclinical models. Synergy potential: pairing iron chelators (e.g., quercetin) with Nrf2 activators (e.g., sulforaphane) may enhance efficacy. Bioavailability challenges: Some compounds (e.g., curcumin, resveratrol) require formulation improvements for optimal delivery.

## Data Availability

The data that support the findings of this study are available from the corresponding authors upon reasonable request.
